# Autoindexing the diffraction patterns from crystals with a pseudotranslation

**DOI:** 10.1107/S0907444909010725

**Published:** 2009-05-15

**Authors:** Nicholas K. Sauter, Peter H. Zwart

**Affiliations:** a Physical Biosciences Division, Lawrence Berkeley National Laboratory, Berkeley, CA 94720, USA

**Keywords:** subgroups, sublattices, cosets, noncrystallographic symmetry

## Abstract

Rotation photographs can be readily indexed if enough candidate Bragg spots are identified to properly sample the reciprocal lattice. However, while automatic indexing algorithms are widely used for macromolecular data processing, they can produce incorrect results in special situations where a subset of Bragg spots is systematically overlooked. This is a potential outcome in cases where a noncrystallographic translational symmetry operator closely mimics an exact crystallo­graphic translation. In these cases, a visual inspection of the diffraction image will reveal alternating strong and weak reflections. However, reliable detection of the weak-intensity reflections by software requires a systematic search for a diffraction signal targeted at specific reciprocal-space locations calculated *a priori* by considering all possible pseudotranslations. Care must be exercised to distinguish between true lattice diffraction and spurious signals contributed by neighboring overlapping Bragg spots, non-Bragg diffraction and noise. Such procedures have been implemented within the autoindexing program *LABELIT* and applied to known cases from publicly available data sets. Routine use of this type of signal search adds only a few seconds to the typical run time for autoindexing. The program can be downloaded from http://cci.lbl.gov/labelit.

## Abstract 2

Lattice patterns containing alternating strong and weak reflections can be identified by a targeted search for the weak signals, permitting a wider range of diffraction patterns to be indexed automatically.

## 1. Introduction

The ability to process a large number of rotation data sets sequentially is a prerequisite for many large-scale projects, including the screening of crystal-growth conditions for optimal diffraction (Page *et al.*, 2005 ▶), the discovery of protein–ligand complexes and the acquisition of multi-crystal data sets involving radiation-sensitive samples. Synchrotron beamlines can facilitate high-throughput work by deploying software packages such as *DNA* (Leslie *et al.*, 2002 ▶) or *Web-Ice* (González *et al.*, 2008 ▶), which present the initial diffraction results in summary form. Under these systems, the underlying computations are automatically delegated to established crystallography programs. This represents an efficiency gain for the end user, who is freed from the burden of managing the data-processing steps separately for each new sample. However, it requires that routine calculations such as autoindexing (the determination of the basis vectors that span the crystal lattice) work flawlessly despite the diversity present in real experimental samples.

To deduce the crystal lattice, many autoindexing algorithms (*e.g.* Kabsch, 1988 ▶, 1993 ▶; Steller *et al.*, 1997 ▶; Sauter *et al.*, 2004 ▶) take the brightest candidate Bragg spots as a starting point. An implicit assumption is that no matter which bright spots are chosen, the subset is representative of the lattice as a whole. This is valid for most macromolecular crystals, as bright spots in each resolution shell are generally distributed randomly; in particular, given some simple prior assumptions about the placement of atoms in the unit cell (Read, 2001 ▶), the probability density of observing an acentric reflection with intensity *I* is
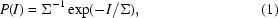
where Σ is the mean reflection intensity of the appropriate resolution shell (Wilson, 1949 ▶; French & Wilson, 1978 ▶). Higher values of Σ are more common in shells of lower resolution.

One exception to this general probability distribution occurs if the structure contains pseudotranslational symmetry (*i.e.* noncrystallographic translational symmetry such that the translational symmetry operator is close to a rational fraction of the cell length; Hauptman & Karle, 1953 ▶, 1959 ▶; Gramlich, 1984 ▶). This causes the reflections to divide into a subgroup of strong intensities and coset(s) of weak intensities. Data of this nature are by no means unusual. Chook *et al.* (1998 ▶), for example, report two crystal structures where the average weak intensity is about 10% of the average strong intensity when considering the lowest resolution shells, in which the disparity between alternating strong and weak intensities is most pro­nounced. Unfortunately, the normal autoindexing strategy is not robust for these cases, as it is not possible to guarantee that a randomly chosen subset of bright reflections will include members of the weak coset. If the chosen reflections are concentrated at low resolution, are few in number and/or if the coset is intrinsically weak, the strong-intensity set dominates. Autoindexing will then produce an (incorrect) model lattice missing the coset altogether, in which the noncrystallographic translation is taken to be an exact crystallographic operation.

This problem would disappear if one could just lower the intensity threshold used to include Bragg spot observations for indexing. Regrettably this does not work consistently in practice, as it is necessary to maintain a high enough cutoff to remove artifacts that would otherwise confuse the indexing algorithm. Instead, we introduce an automated procedure that is meant to emulate the empirical process reported by various groups (*e.g.* Warkentin *et al.*, 2005 ▶). Firstly, the data set is autoindexed normally to produce a presumptive basis set that may or may not span the coset of weak reflections, if any is present. The raw data are then re-examined to ascertain whether there is additional Bragg diffraction in between positions on the modelled lattice. If so, the presumptive basis vectors are transformed accordingly, producing a new lattice model spanning both the strong and the weak reflections. When implemented within the autoindexing pro­gram *LABELIT* (Sauter *et al.*, 2004 ▶), this approach takes only a few extra seconds of computational time and identifies cases of pseudotranslation with high fidelity. The procedure has the additional benefit of being able to identify the presence of pseudo-translational symmetry at the stage of autoindexing, in contrast to Patterson methods, which rely on the availability of reasonably complete data (Zwart *et al.*, 2005 ▶).

## 2. Mathematical background

Autoindexing gives a complete description of the presumptive reciprocal lattice **L** and its relation to the laboratory coordinate system in the form of an orientation matrix
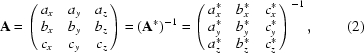
where the matrix components are the orthonormal projections of the unit-cell basis vectors **a**, **b**, **c** (and reciprocal-cell basis vectors a*, b*, c*) that have been converted to reduced form (Grosse-Kunstleve *et al.*, 2004 ▶). The presence of pseudotranslation, associated with alternating weak Bragg spots that are not on the lattice **L**, leads to the identification of the true reciprocal lattice **L**′ given by the orientation matrix
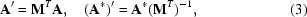
where **M** is a transformation matrix whose integer determinant *n* is the ratio of unit-cell volumes, *n* = |**A**′|/|**A**|. Using the terminology of Rutherford (2006 ▶), we call **L**′ a sublattice of **L** and *n* the index of the sublattice. Although there are an infinite number of index-*n* sublattices of **L**, a key result from group theory (Billiet & Rolley Le Coz, 1980 ▶) is that the number of distinct sublattices is finite and small. Unit-cell doubling, for example, leads to only seven unique sublattices: those with doubled **a**, **b** or **c** basis vectors, those with pseudo *A*-, *B*- or *C*-face centering and one with pseudo body-centering. Table 1 ▶ shows the upper-triangular matrices **M** and transformed basis vectors associated with each of these cases.

Borrowing nomenclature from group theory, this paper uses the term coset to refer to the weak reflections on the sublattice that are not part of the main lattice **L**. Reciprocal-lattice vectors form an Abelian group under the operation of vector addition, with **L** being a subgroup of **L**′. The coset decomposition of **L**′ with respect to **L**,
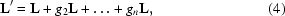
identifies cosets (or subsets) *g*_2_ **L**, …, *g*_*n*_ **L** obtained by adding the vectors *g*_2_, …, *g*_*n*_ to each vector of **L**. For example, the doubling of unit-cell vector **a** leads to a single coset with *g*_2_ = ½a* and its tripling leads to cosets with *g*_2_ = 1/3a* and *g*_3_ = 2/3a*.

## 3. Computational approach

It is straightforward to enumerate all distinct transformations **M** that give sublattices of index *n* (Billiet & Rolley Le Coz, 1980 ▶; Zwart *et al.*, 2006 ▶). Having performed this, the following algorithm is used to detect sublattices in the raw data. After autoindexing to determine **A**, perform a loop over all matrices **M** to give **A**′. For each **A**′ and for each rotation photograph used in autoindexing (*LABELIT* normally uses two 1° rotations positioned 90° apart in ϕ), predict the positions of all reflections on the detector out to a certain resolution limit. For each reflection with Miller index **h**′, back-transform the Miller index into the original (**L**) reciprocal basis,
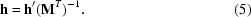
Miller indices are then divided into two sets. Those with all-integer **h** components ({**h**_integer_}) are spanned by the main lattice **L**, while those containing a fractional **h** component ({**h**_fractional, **M**_}) are associated only with the sublattice **L**′. Focusing exclusively on this latter coset, the raw data are investigated to see if there is (weak) Bragg scattering at these predicted spot positions. If so, it is concluded that the correct lattice is **L**′. After the loop over all matrices **M** is finished, the final orientation matrix (**A**′ if a sublattice has been discovered, otherwise **A**) is analyzed to determine the metric symmetry as previously described (Sauter *et al.*, 2004 ▶, 2006 ▶).

**Figure 1:**
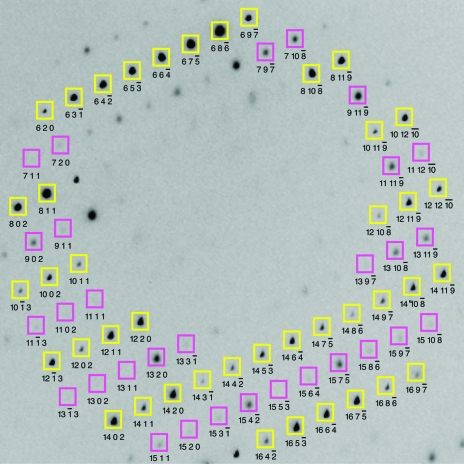
Detail of a 1° rotation photograph taken from the data set used for solving PDB entry 2qyv. Yellow boxes show the presumptive lattice if the image is indexed based on the brightest spots only, ignoring the weak signal arising from pseudotranslational symmetry. Magenta boxes are additional lattice positions predicted by one of the seven possible cell-doubling transformations (doubling of **a**) listed in Table 1 ▶. The yellow and magenta boxes together produce the lattice of the published structure.

This approach completely avoids the original dilemma of lowering the spot-picking threshold sufficiently to sample the sublattice, which carries the risk of introducing artifacts. Instead, we target the sublattice search at specific detector positions and thus can detect weak signals down to very low signal-to-noise levels. Fig. 1 ▶ shows the detection of a sublattice with *n* = 2.

Since the signal of interest is inherently of low intensity, it is necessary to carefully eliminate phenomena that could be falsely interpreted as Bragg scattering from a sublattice coset. Such decoy signals are treated in §§3.1–3.3 [Sec sec3.2] [Sec sec3.3].

### 3.1. Rejection of intensity outliers

**Figure 2:**
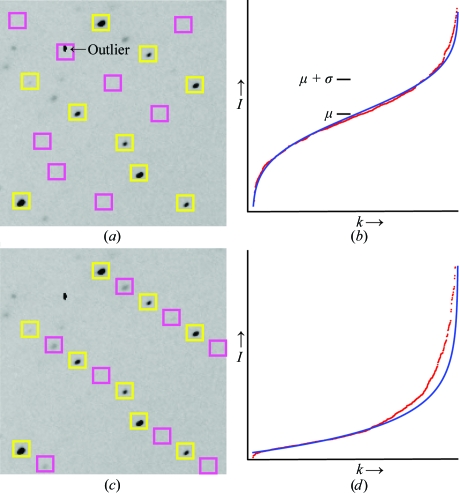
Analysis of a 1° rotation photograph from the 2qyv data set. Twofold pseudotranslational symmetry produces a main lattice of bright reflections (yellow boxes) and a coset of weak reflections (magenta boxes) that is identified incorrectly and correctly in (*a*) and (*c*), respectively. The correct cell-doubling transformation can be selected by measuring the intensities *I* at predicted coset spot positions, but a potential pitfall of this calculation is illustrated by the single outlier that heavily biases the average intensity 〈*I*〉 in (*a*). The difficulties are resolved by modeling the entire population as either a Gaussian distribution indicative of measurement noise (*b*) or an approximate exponential distribution indicative of real Bragg diffraction (*d*). Red dots in (*b*) and (*d*) are intensity observations from the cosets depicted in (*a*) and (*c*) respectively, in the 8–3 Å resolution range, plotted against sequence number *k*, where the set of intensities has been re-sorted by increasing value. Blue curves show the best-fit expected intensities, after outlier rejection, for Gaussian or exponential distributions as in (8) and (11).

One potential pitfall arises from the undesirable presence of outlying pixel intensities in the raw data caused by ice crystals, zingers or other processes (Bourgeois, 1999 ▶). An example is seen in Fig. 2 ▶(*a*), in which a small group of saturated CCD pixels occurs by chance at the Bragg spot position predicted by pseudo-body centering. A naïve method for confirming pseudotranslation would be to calculate the average center-pixel intensity 〈*I*〉 over the coset of predicted spots {**h**_fractional, **M**_}. Unfortunately, this simple metric produces a false result in the case of Fig. 2 ▶(*a*) as the single outlying spot biases 〈*I*〉 enough that the incorrect pseudo body-centered lattice scores higher than the correct lattice shown in Fig. 2 ▶(*c*). In order to reliably analyze the data, one must consider the intensity distribution over the entire coset population. Disregarding the single outlier, coset intensities measured on the incorrect sublattice are distributed on a Gaussian profile (Fig. 2 ▶ *b*) as expected from background noise. In contrast, coset intensities from the correct sublattice form an approximate exponential distribution (Fig. 2*d*) consistent with Bragg diffraction [equation (1); Gramlich, 1984 ▶]^1^.

To implement population modeling in software, the raw data are initially conditioned by removing the background signal of the image as described previously (Zhang *et al.*, 2006 ▶). The background is modelled on a 50-pixel grid, and we improve upon the previous work by treating the background within each grid area as an inclined (rather than a flat) plane, deriving the local plane constants by the method of Rossmann (1979 ▶). After subtraction of the background, pixel values are re-expressed in terms of the background variance (specifically, in units of the root-mean-squared deviation of local background pixels away from the best-fit background plane). We then take the set of center-pixel intensity measurements at predicted Bragg spot positions (within a suitably thin resolution shell) and attempt to fit the population to both a Gaussian distribution
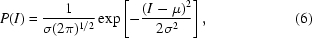
with mean μ and standard deviation σ, and to an exponential distribution as in (1). Outliers are rejected by random-sample consensus (Fischler & Bolles, 1981 ▶). Briefly, model parameters (μ and σ for a Gaussian distribution; Σ for an exponential distribution) are calculated from a very small randomly chosen subset of the population. This process is repeated a large number of times, allowing the selection of a final model and a final distribution type (Gaussian or exponential) that fits the largest number of data from the whole set. The criteria for evaluating model fit are explained in Appendix *A* [App appa]. The method is useful for distinguishing Bragg diffraction from noise, even though the analysis is performed before the data-integration step and before Lorentz, polarization and partiality corrections are applied to improve the accuracy of the potential Bragg intensities.

### 3.2. Rejection of nonconforming spot profiles

**Figure 3:**
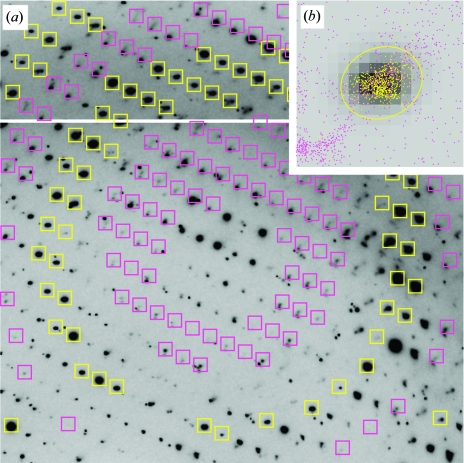
The use of positional information in a diffraction pattern to rule out the presence of pseudotranslation. In (*a*), yellow and magenta boxes are centered at Bragg positions on the main lattice and candidate coset, respectively. (*b*) plots the observed position of the pixel maximum found in each box in relation to the predicted box position. The plot shows that spot maxima on the main lattice (yellow dots) cluster tightly around their predicted positions. The distribution can be modeled as a bivariate Gaussian, with the yellow ellipse enclosing 95% of the probability density. Spot maxima on the candidate coset (magenta dots) lie mainly outside of this ellipse (70% in this case), showing that the coset spots do not conform to the expected sublattice.

In addition to testing whether the intensity distribution is consistent with Bragg diffraction, it is also necessary to confirm that the observed spot positions match the candidate sublattice to high precision. This guards against unwarranted conclusions from diffraction patterns such as that shown in Fig. 3 ▶. Here, the Bragg spots on the main lattice {**h**_integer_} are round in shape and are perfectly centered at their predicted positions. However, while the spot intensities on the candidate coset {**h**_fractional, **M**_} form an acceptable exponential distribution, the spot shapes appear to be broken and are not well centered on the lattice. Rather than being an indication of pseudotranslation, this diffraction pattern is likely to arise from some other phenomenon such as fragmentation of the crystal sample.

The automatic rejection of this candidate sublattice is accomplished by a statistical analysis of spot positions. The brightest spots used for autoindexing are grouped together to form an average spot profile, after which a rectangular mask is constructed that accommodates the profile plus a strip of background pixels on each side. Fig. 3 ▶(*b*) shows a 16 × 16 pixel mask with greyscale shading to indicate the normalized intensity of each profile pixel. The mask is now positioned on the image at every Miller index **h**′ (Fig. 3 ▶ *a*) and the location of the strongest observed pixel within the mask is noted. This is performed separately for the main lattice {**h**_integer_} and candidate coset {**h**_fractional,** M**_}. In order for the candidate coset to be accepted as valid, the coset pixel maxima must be clustered normally around the predicted spot positions, just as they are for the main lattice. Bivariate Gaussian statistics are used to model the population of pixel maxima from the main lattice, and the candidate sublattice is rejected if too many of the coset maxima (*e.g.* > 50%) fall outside this distribution, as in Fig. 3 ▶(*b*).

### 3.3. Avoidance of overlapping Bragg reflections

**Figure 4:**
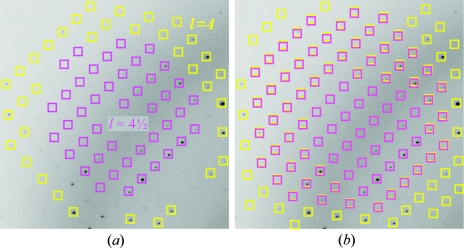
Detail from a 1° rotation photograph used to solve PBD entry 1vr9. (*a*) shows an initial lattice model from autoindexing, with effective mosaicity *m* = 0.3°. The apparent presence of a lattice layer with half-integer Miller indices *l* = 4½ (magenta) would seem to be consistent with **c**-axis doubling. However, these Bragg spots can also be interpreted as arising from highly mosaic rocking curves from the main lattice layers at *l* = 4 (yellow) or *l* = 5 (not shown). The overlap of integer- and half-integer-layer spots upon increasing the mosaicity parameter [*m* = 1.3° is depicted in (*b*)] prevents the use of these particular spots as evidence of pseudotranslation.

The above examination of Bragg spots relies on the assumption that lattice positions are well separated across the detector face. Yet it is understood (Dauter, 1999 ▶) that factors such as large unit cell and high mosaicity will inevitably produce spot overlap. Therefore, before any signal analysis is performed, pairs of **h**_integer_ and **h**_fractional,**M**_ Bragg spots are removed if there is mutual overlap of the masks described in §[Sec sec3.2]3.2 (and depicted as boxes in Figs. 1–4). This is performed separately for each sublattice **L**′ and the sublattice is rejected as a candidate if the remaining non-overlapped spots are too few in number for meaningful statistics (*e.g.* < 200). The efficient identification of overlapping masks is facilitated by the use of the Approximate Nearest Neighbor software library (Arya *et al.*, 1998 ▶).

Discovery of spot overlaps relies on the accurate ability to predict whether particular Miller indices will be in diffracting position for a given rotational setting of the crystal. In this context it is important to consider two limitations. Firstly, the lattice parameters used here (including the orientation matrix **A** and the effective mosaicity *m*) are only initial estimates derived from autoindexing. The parameters are post-refined in a subsequent data-processing step (Winkler *et al.*, 1979 ▶; Rossmann *et al.*, 1979 ▶) after integration, but the post-refined values are not yet available at the step utilized here for considering pseudotranslation. Furthermore, the use of a single effective mosaicity parameter *m* is a simplification that does not account for separate contributions from different physical sources of crystal dis­order, anisotropic disorder or the distinct effects of beam divergence. We do not attempt to create a highly accurate or detailed model, and consequently must allow for the possibility that the diffraction intensity at the position of a candidate coset spot might actually arise from the rocking-curve tail of a nearby main-lattice spot that is not predicted to diffract based on the available model.

This safeguard is implemented by adding a second overlap-detection step. Coset spots **h**_fractional,**M**_ are individually considered, enumerating all Miller indices on the main lattice **h**_integer_ that are immediately adjacent in reciprocal space. Detector positions are calculated for each of these **h**_integer_ spots, even if the crystal rotational setting needed to satisfy the reflecting conditions (given the available model parameters **A** and *m*) is outside the rotational range used to acquire the image. In this way, we can reject coset spots that could potentially be overlapped if the model value *m* is unrealistically small (Fig. 4 ▶). Enumeration of all neighboring spots is computationally intensive, so the calculation is limited to a small set of representative coset spots distributed across the face of the detector. Candidate coset spots are rejected if the nearest representative spot is potentially overlapped.

### 3.4. Sublattice validation using integrated intensities

The strategy outlined above examines individual pixel intensities in the raw data to detect any sublattice that may have been ignored during the autoindexing step. To verify the presence of a sublattice, it is useful to re-examine the Bragg intensities after the data have been integrated, as other authors have done (*e.g.* Chook *et al.*, 1998 ▶). To implement this, the raw data (typically the one or two rotation images that have been used for autoindexing) are integrated with *MOSFLM* (Leslie, 1999 ▶) based on the triclinic basis set **A**′. For each potential transformation matrix **M**, the Miller indices **h**′ of the integrated data are then back-transformed by (5), dividing the intensities into a main set with indices {**h**_integer,**M**_} and a coset with indices {**h**_fractional,**M**_}. A particular pseudotranslation is inferred if both (i) the coset has significant data, *i.e.* the coset’s average intensity-to-error ratio 〈*I*/σ(*I*)〉 is greater than 1.0, and (ii) the average intensities on the main lattice 〈*I*_M_〉 are significantly greater than those on the coset 〈*I*_C_〉, at least in the lowest resolution bins. These calculations can be performed on single images (see Table 2 ▶), offering the potential for pseudotranslation to be validated prior to acquiring the complete data set.

## 4. Application to experimental data

The public availability of an archive of complete diffraction data sets from the Joint Center for Structural Genomics (Burley *et al.*, 2008 ▶; http://www.jcsg.org) provides an excellent opportunity to test new methods on real data (Baker *et al.*, 2008 ▶). Table 2 ▶ illustrates two cases where pseudotranslation is clearly detected by examining individual rotation images.

JCSG’s published structure of *Haemophilus somnus* Xaa-His dipeptidase (PDB entry 2qyv) is based on 90° wedges of rotation data taken at four X-ray wavelengths from a single crystal with space group *P*2_1_2_1_2 and unit-cell parameters *a* = 174, *b* = 84, *c* = 123 Å. The asymmetric unit contains two protomers related by a pseudo-crystallographic translation, as evidenced (for example) by the presence of a strong peak at the fractional coordinates (½, ½, ½) of a native Patterson map. It is possible to detect a pattern of alternating strong and weak Bragg spots on each image, but the ability to reliably choose the correct lattice during autoindexing is completely dependent on the details of the spot-picking procedure. In this particular data set, the spot-picking program *DISTL* (Zhang *et al.*, 2006 ▶) typically detects over 1000 candidate Bragg spots per image. If this entire set is used, separate autoindexing of each image always produces the correct *P*2_1_2_1_2 lattice because the weak coset is always adequately represented. However, using all the candidate spots generally carries the risk that the lattice will be obscured by spurious signals that only appear to be Bragg spots; therefore, autoindexing success is often improved by fitting the lattice to a smaller subset consisting of the brightest spots. For the 2qyv data set, a safer algorithm (using the 300 brightest spots on each image for autoindexing) completely misses the alternating weak spots, invariably producing basis vectors consistent with an *I*-centered ortho­rhombic lattice with unit-cell parameters *a* = 84, *b* = 123, *c* = 174 Å, where the asymmetric unit contains only one protomer. The situation is reconciled by applying the method­ology of §[Sec sec3]3 (see Figs. 1 ▶ and 2 ▶, and Table 2 ▶), allowing us to validate the presence of alternating weak spots after using the robust autoindexing method with the brightest spots.

JCSG’s structure of *Thermotoga maritima* aspartate aminotransferase (PDB entry 2gb3) provides a contrasting example where the normal autoindexing approach is sufficient to detect pseudotranslation. Here, the space group is *P*2_1_ with unit-cell parameters *a* = 75, *b* = 214, *c* = 77 Å, β = 112°. The asymmetric unit contains three α_2_ dimers related by threefold pseudo-crystallographic translation, as shown by the presence of a strong peak at the fractional coordinates (0, ⅓, 0) of a native Patterson map. In this case, the safe method (sorting the candidate Bragg spots and indexing on the brightest ones) does not limit the ability the choose the correct lattice. In all the images archived (86° wedges composed of 0.25° rotation images taken at two X-ray wavelengths and 130° wedges composed of 1° rotation images taken at three X-ray wavelengths), as long as enough candidate Bragg spots are used to produce any lattice solution, the correct sublattice is found without the additional computational steps taken in §§3.1–3.3 [Sec sec3.2] [Sec sec3.3] (see Table 2 ▶).

## 5. Software availability

The procedures described here are included in *LABELIT* v.1.1 (and above), which is available for download by non­commercial users at http://cci.lbl.gov/labelit. A benefit of the present treatment is that pseudotranslation can be detected from a single image, automatically and without visual inspection, immediately after the autoindexing step. It is therefore possible for the results to play a role in decision-making during data aquisition, if for example the identification of the correct Bravais lattice permits an advantageous choice of data-collection strategy (Dauter, 1999 ▶).

Adjustments to the exact algorithm used can be made by setting run-time parameters, potentially assisting with the analysis of difficult cases where the default settings do not produce the desired result. Sample parameters are listed in Table 3 ▶ and a complete listing is given in the online documentation.

## Figures and Tables

**Table 1:** Transformations giving the sublattices of index *n* = 2 (unit-cell doubling)

Type of unit-cell doubling	Transformation matrix **M**	Sublattice basis **a**′, **b**′, **c**′	Miller indices **h**′ of weak reflections in the sublattice	Peak position in the sublattice Patterson function
Doubling of **a**	(200 010 001)	2**a**, **b**, **c**	*h*′ odd	½, 0, 0
Doubling of **b**	(100 020 001)	**a**, 2**b**, **c**	*k*′ odd	0, ½, 0
Doubling of **c**	(100 010 002)	**a**, **b**, 2**c**	*l*′ odd	0, 0, ½
*C*-face centering	(210 010 001)	2**a**, **b** + **a**, **c**	*h*′ + *k*′ odd	½, ½, 0
*B*-face centering	(201 010 001)	2**a**, **b**, **c** + **a**	*h*′ + *l*′ odd	½, 0, ½
*A*-face centering	(100 021 001)	**a**, 2**b**, **c** + **b**	*k*′ + *l*′ odd	0, ½, ½
Body centering	(211 010 001)	2**a**, **b** + **a**, **c** + **a**	*h*′ + *k*′ + *l*′ odd	½, ½, ½

**Table 2:** Representative diffraction statistics indicative of pseudotranslation *I* is the integrated intensity and σ is the experimental error. *I*_M_ are main lattice intensities and *I*_C_ are sublattice coset intensities. *N* is the number of observations averaged in each resolution range. The intensity data represent partially and fully measured reflections from a single 1° rotation image after application of Lorentz, polarization and partiality corrections.

	PDB entry 2qyv							PDB entry 2gb3						
	Main lattice			Sublattice coset				Main lattice			Sublattice coset			
Resolution (Å)	〈*I*〉	〈*I*/σ(*I*)〉	*N*	〈*I*〉	〈*I*/σ(*I*)〉	*N*	〈*I*_M_〉/〈*I*_C_〉	〈*I*〉	〈*I*/σ(*I*)〉	*N*	〈*I*〉	〈*I*/σ(*I*)〉	*N*	〈*I*_M_〉/〈*I*_C_〉
∞–7.9	14437	19.5	46	273	9.1	45	52.9	10167	18.1	30	325	8.8	45	31.3
7.9–5.6	4371	13.6	78	304	8.5	94	14.4	1876	11.7	35	303	6.3	92	6.2
5.6–4.6	6449	12.6	119	755	9.5	117	8.5	2863	10.3	45	578	5.6	111	5.0
4.6–4.0	6506	12.5	120	883	8.7	138	7.4	2769	9.0	59	704	5.5	141	3.9
4.0–3.5	4331	11.5	164	743	7.9	154	5.8	1977	7.8	87	545	4.4	142	3.6
3.5–3.2	2724	9.2	160	547	5.8	178	5.0	1090	5.8	91	297	2.8	168	3.7
3.2–3.0	1577	8.0	182	375	4.4	176	4.2	503	3.5	97	280	2.5	179	1.8
3.0–2.8	921	6.8	185	233	3.2	193	4.0	308	2.6	102	258	2.3	195	1.2
2.8–2.6	615	5.5	165	185	2.7	168	3.3	276	2.1	99	169	1.6	213	1.6
2.6–2.5	323	4.1	118	90	1.7	125	3.6	148	1.3	115	132	1.3	207	1.1

**Table 3:** Sample command-line parameters affecting sublattice detection

Parameter (with default value)	Function
sublattice_allow = True|False (True after v.1.1; False previously)	Turn on sublattice detection as described in §3
sublattice_maximum_modulus = 3	Maximum value of *n*, the sublattice index
sublattice_pdf_file = <filename> (default: no output)	Create a graphical representation of the sublattice as in Fig. 1 ▶.

## Appendix A Parameter fitting

Given a Gaussian distribution with mean μ and standard deviation σ as in (6), the cumulative distribution function, or probability that an observation will have a value between −∞ and *I*, is

where erf is the error function. As a result, if there are *N* observations sorted in order of increasing value and indexed by the symbol *k* (*k* = 0, 1, …, *N* − 1), the expected intensity of the *k*th value can be modeled as

In §[Sec sec3.1]3.1 we reject the *k*th observation *I*_obs_(*k*) as an outlier if the difference between observation and expectation is too large, *e.g.*

A computationally tractable approximation for the erf^−1^ function is given by Winitzki (2008 ▶), which while low in precision is sufficient for the present application.

For exponential distributions with mean value Σ as in (1) [Sec sec1], the cumulative distribution function is

The expected intensity of the *k*th value in a set of *N* observations is

where *b* is an *ad hoc* parameter allowing the exponential decay in the distribution of intensities to begin at a nonzero value. Here, a useful criterion for rejecting outliers is

## Footnotes

1 The intensity distribution of (1) applies only to acentric reflections, not to centric reflections (Wilson, 1949 ▶; French & Wilson, 1978 ▶). However, since the Bravais symmetry has not yet been identified at this stage of the analysis, we do not strictly know which measurements arise from acentric or centric Bragg spots. Here, we take the simple expedient of treating all observations as if they arise from acentric reflections, relying on the prevalence of acentric spots to allow the population to be modelled according to (1).
